# First live birth after *in vitro* fertilization in a woman with Alström syndrome: a case report

**DOI:** 10.3389/frph.2025.1585308

**Published:** 2025-07-15

**Authors:** Andrea Roberto Carosso, Marco Carosso, Alberto Revelli, Stefano Canosa, Luca Marozio, Gianluca Gennarelli, Pietro Maffei, Serena Ditaranto, Chiara Benedetto

**Affiliations:** ^1^ Obstetrics and Gynecology 1U, Physiopathology of Reproduction and IVF Unit, Department of Surgical Sciences, Sant'Anna Hospital, University of Turin, Turin, Italy; ^2^ Gynecology and Obstetrics 1U, Departments of Surgical Sciences, University of Turin, Turin, Italy; ^3^ Division of Gynecology and Obstetrics 2, Department of Surgical Sciences, City of Health and Science, University of Turin, Turin, Italy; ^4^ IVIRMA, Global Research Alliance, LIVET, Turin, Italy; ^5^ Department of Medicine, University of Padua, Padua, Italy; ^6^ Department of Anesthesiology and Intensive Care, A.O.U. Città Della Salute E Della Scienza Di Torino, Turin, Italy

**Keywords:** Alström syndrome, *in vitro* fertilization, pregnancy, ICSI, preimplantation genetic diagnosis

## Abstract

**Purpose:**

Alström syndrome (AS) is an extremely rare, autosomal recessive genetic disorder. Fertility implications are particularly relevant for women affected by AS, and no cases of patients achieving pregnancy and live birth with *in vitro* fertilization (IVF) have been previously described. This case report describes the first worldwide live birth after IVF in a woman affected by AS.

**Methods:**

This case report describes the IVF procedure and pregnancy management of an infertile woman suffering from AS.

**Results:**

After two intracytoplasmic sperm injections (ICSI), combined with preimplantation genetic testing for aneuploidies (PGT-A), a euploid blastocyst was transferred, resulting in a clinical pregnancy. Gestation was complicated by gestational diabetes mellitus and chronic hypertension, which were well controlled by specific treatments. At 38 weeks of gestation, a healthy child of 3,110 g was born.

**Conclusion:**

The successful outcome of this rare case suggests that IVF is feasible in the case of women with AS, but a multidisciplinary, continuous follow-up aimed at controlling comorbidity-linked complications is needed.

## 1 Introduction

Alström syndrome (AS) is an extremely rare, autosomal recessive genetic disorder with an estimated prevalence of 1 in 100,000–1,000,000 individuals (1). It is caused by genetic alterations of the *ALMS1* gene, located on chromosome 2 at the position p13.1. A series of disease-causing variants in exons 8 (49%), 10 (17%), and 16 (19%) of the *ALMS1* gene were described (2). ALMS1 protein is widely expressed. Although its function is not fully known, it seems to be involved in the structure and function of ciliated cells and to affect ciliary movement (3).

AS may appear with two distinct phenotypes: typical and mild. In the typical form, the patient is affected by cone-rod dystrophy, hearing loss, severe insulin resistance (IR), type 2 diabetes mellitus, non-alcoholic fatty liver disease, cardiomyopathy, progressive renal and liver failure, hypothyroidism, hyperandrogenism (in females), or hypogonadism (in males). The clinical picture appears in early infancy and may be very variable as for severity and onset. The mild form has been recently described: It is characterized by the slow onset of visual impairment and photophobia, preserved or mildly impaired hearing, and mild metabolic complications. Some studies suggest that patients with the mild phenotype carry a variant with a genetic alteration of the *ALMS1* gene located at a peculiar position, just upstream of exon 7. In general, however, there is no clear correlation between the site of genetic alteration and the phenotype (4, 5).

Fertility implications are particularly relevant for women affected by the typical AS variant. They usually have a combination of signs and symptoms such as hyperandrogenism, oligomenorrhea, precocious puberty, polycystic appearance of the ovary, and endometriosis. They may also exhibit abnormal breast development while maintaining normal external genitalia, uterus, and fallopian tubes (1). Insulin resistance with hyperinsulinemia may lead to a complete alteration of ovarian steroidogenesis, hyperandrogenism, and impairment of follicular development, with chronic anovulation developing in a way similar to that occurring in polycystic ovary syndrome (PCOS) (6). Different from PCOS, however, these patients have a higher risk of premature ovarian insufficiency, as in some cases postmortem analysis revealed extensive ovarian fibrosis and poor follicular reserve (7).

Assisted reproductive technologies (ART) treatments include a series of medical procedures aimed at infertility care, but also techniques that allow for screening and deselecting embryos with genetic abnormalities by preimplantation genetic testing (PGT), either for monogenic diseases (PGT-M) or aneuploidies (PGT-A). *In vitro* fertilization (IVF) and PGT-M can be used for the prevention of AS transmission to the offspring.

To date, there are no published cases of AS patients achieving pregnancy and live birth with IVF, and herein we report the first case.

## 2 Case description

We observed a 32-year-old nulliparous woman affected by the mild form of Alström syndrome, with a homozygous non-sense substitution in exon 5. Her genotype was ALMS1c.[1046G>A]+[1046G>A]p.(Trp349*)+(Trp349*) (2), a variant, sometimes observed in cases with retinal dystrophy during childhood (8), that was also expressed in the patient's sister, with both parents being heterozygote carriers.

When the patient came to our observation, her body mass index (BMI) was 27, her body weight was 65 kg, and she had regular menstrual cycles with multi-follicular US appearance of the ovaries.

Her symptoms included photophobia with decreased vision, hypoacusia needing hearing prosthesis, fatty liver with increased serum levels of transaminases, and chronic hypertension with normal heart ejection fraction. The prepregnancy biochemical and hormonal assessments are presented in Table 1.

**Table 1:** Prepregnancy biochemical, metabolic, and hormonal test results.

Blood tests	Prepregnancy	Normal value
Fasting glucose (mmol/L)	103	70–100
Fasting insulin (mU/L)	12.2	3.2–16.3
Fasting C-peptide (ug/L)	4.35	0.80–4.2
Glycated hemoglobin (mmol/mol)	29	20–42
Total cholesterol (mg/dl)	196	<200
Low-density lipoprotein cholesterol (mg/dl)	133	<130
High-density lipoprotein cholesterol (mg/dl), low risk	42	>60
Triglycerides (mg/dl)	89	50–175
Uric acid (mmol/L)	0.33	0.15–0.35
Creatinine (mg/dl)	0.57	0.55–1.02
Aspartate transaminase (U/L)	56	<35
Alanine transaminase (U/L)	161	<35
Alkaline phosphatase (U/L)	125	33–98
Gamma-glutamyltransferase (UI/L)	316	3–45
Alpha amylase (U/L)	37	13–53
Testosterone (nmol/L)	2.91	0.52–2.43
Dihydrotestosterone (nmol/L)	2.08	0.08–1.26
Estradiol (pmol/L)	134	
Dehydroepiandrosterone sulfate (umol/L)	5.80	0.90–9.21
Follicle-stimulating hormone (mIU/ml)	5.2	1–8–8
Luteinizing hormone (mIU/ml)	6.6	1–18
Prolactin (ng/ml)	8.4	2–29
Sex hormone-binding globulin (nmol/L)	43.9	18.0–144
Androstanediol glucuronide (ug/L)	4.80	0.34–7.53
Androstenedione (nmol/L)	8.1	0.75–3.89
Cortisol (nmol/L)	294	185–624
Free thyroxine (pmol/L)	14.16	9–22
Thyroid-stimulating hormone (mIU/L)	1.50	0.3–5
Anti-Müllerian hormone (pg/L)	2.5	

The couple arrived to us after an infertility period of 2 years, during which she often used urinary tests for the detection of the LH surge to target intercourse. Even if not always positive, most of the months had a positive urinary LH detection, suggesting the occurrence of ovulation.

Hormonal tests aimed at evaluating the ovarian reserve (Table 1) and an examination of the partner's semen were requested. The semen showed a sperm concentration of 6.8 million/ml, 28% progressively motile sperm, and 2% normal morphology, indicating oligo-terato-astheno-zoospermia likely responsible for infertility, together with the inconstant ovulation. In view of these results, intracytoplasmic sperm injection (ICSI) was planned. The genetic evaluation of the male partner showed that he was not a healthy carrier of AS, and the risk of getting an ill offspring was <1/15,000; for this reason, PGT-M to search for mutations on the embryo's *ALMS1* gene was not considered.

The first ICSI cycle started with controlled ovarian stimulation (COS) with human menopausal gonadotropin (Menopur, Ferring, Saint-Prex, Switzerland), 150 IU/day, starting the first day of menstruation, with the addition from Day 5 of GnRH antagonist ganirelix (Orgalutran fi, Merck Sharp & Dohme, Kenilworth, NJ, USA). COS was monitored by serial transvaginal US and serum estradiol (E2) measurements performed every second day from stimulation day and continuing until at least two follicles reached 18 mm mean diameter. At this point, ovulation was triggered by injecting 0.2 mg triptorelin (Decapeptyl, Ferring, Saint-Prex, Switzerland). US-guided transvaginal oocyte pickup (OPU) was performed approximately 36 h after trigger, under local anesthesia (paracervical block) and mild sedation. Nine cumulus–oocyte complexes (COCs) were retrieved, washed in buffered medium (gamete medium, Vitrolife, Sweden), and within 2 h from OPU, oocytes and cumulus cells were separated by gently pipetting in 600 µl buffered medium containing hyaluronidase (HYASE-10X, Vitrolife, Sweden) obtaining six metaphase II (MII) oocytes. The semen sample was processed by density gradient centrifugation in order to select motile, morphologically normal spermatozoa. Conventional ICSI was performed on all mature oocytes within 4 h after oocyte collection. The following day, normal 2PN fertilization was confirmed on all injected oocytes. Six zygotes were cultured in 700 µl cleavage medium (Vitrolife, Sweden) overlaid with 550 µl of mineral oil (LifeGuard Oil, LifeGlobal IVF, Denmark) up to development Day 3. At this stage, the culture medium was changed into stage-specific medium (Sequential Blast, Origio, Ireland) until the fully expanded blastocyst stage. Embryo culture was carried on in a controlled humidified atmosphere (37°C, 6% CO_2_) with low oxygen tension in BT37 benchtop incubators (Planer, Origio, Ireland). Two viable blastocysts were obtained on Day 6 and were submitted to trophectoderm biopsy with laser-assisted zona opening for PGT-A. Vitrification was performed within 30 min after trophectoderm biopsy with the Cryotop carrier vitrification kit (Kitazato, Japan). Comprehensive chromosomal testing on trophectoderm cells was performed by next-generation sequencing (NGS) (9) by Igenomix (Marostica, Italy). The method was designed to specifically identify constitutive whole-chromosome meiotic aneuploidies. Both biopsied blastocysts resulted to be aneuploid.

The second ICSI cycle was carried out with COS using recombinant FSH plus LH 2:1 ratio (Pergoveris, Merck Sharp & Dohme, Kenilworth, NJ, USA), starting the first day of menstruation with 150 UI/day; the GnRH antagonist was added from Day 5 onward. After GnRH-analog trigger, 15 oocytes were retrieved, of which 12 were nuclearly mature. Conventional ICSI was performed on all mature oocytes within 4 h after oocyte collection. All injected oocytes showed normal fertilization and grew regularly until Day 3 of *in vitro* culture; four viable blastocysts were obtained on Day 6 and underwent trophectoderm biopsy for PGT-A. Three blastocysts resulted in euploidy. Two months later, a single euploid blastocyst was transferred in a modified spontaneous cycle: When the spontaneously developed preovulatory follicle reached 18 mm in diameter, ovulation was triggered by subcutaneous administration of highly purified chorionic gonadotropin (hCG, Gonasi HP 5000, IBSA, Lugano, Switzerland). Unfortunately, pregnancy was not obtained. The next month, a second embryo transfer was performed with the same protocol, and after 12 days, a positive pregnancy test was obtained. Pregnancy was confirmed after 2 weeks with a US scan.

A thorough clinical follow-up of maternal and fetal conditions was carried out throughout pregnancy by clinical check plus US examination (Table 2). Overall, obstetrical and neonatal outcomes are reported in Table 3.

**Table 2:** Pregnancy biochemical, metabolic, and hormonal test results.

Blood tests	First trimester	Second trimester	Third trimester	Normal value
Hemoglobin (g/L)	154	145	129	123–153
Fasting glucose (mmol/L)	98	90	117	70–100
Oral glucose tolerance test				
Basal glucose	93			<92
Glucose60′	190			<180
Glucose120’ (mmol/L)	150			<153
Fasting insulin (mU/L)	8.2	4.2	5.5	3.2–16.3
Fasting C-peptide (ug/L)	3.4	2.6	3.8	0.80–4.2
Glycated hemoglobin (mmol/mol)	24	28	30	20–42
Total cholesterol (mg/dl)	226	210	198	<200
Low-density lipoprotein cholesterol (mg/dl)	132	128	130	<130
High-density lipoprotein cholesterol (mg/dl)	72	65	70	>60
Triglycerides (mg/dl)	89	90	85	<150
Uric acid (mmol/L)	4.4	4.5	6.3	0.15–0.35
Creatinine (mg/dl)	0.6	0.60	0.56	0.55–1.02
Aspartate transaminase (U/L)	22	17	18	7–35
Alanine transaminase (U/L)	33	20	21	10–35
Alkaline phosphatase (U/L)	54	61	63	33–98
Gamma-glutamyltransferase (U/L)	43	33	9	3–45
Alpha amylase (U/L)	40	38	30	13–53
Urinary protein (g/L)	0.18	0.21	0.20	<0.3
Thyroid-stimulating hormone (mIU/L)	2.07	2.11	2	0.3–5

**Table 3:** Baseline characteristics of the patient and obstetric and neonatal outcomes.

Parameter	Clinical finding
Weight before pregnancy (kilograms)	65
Weight at term of pregnancy	72
Height (cm)	155
Comorbidities	Chronic hypertension, photophobia with decreased vision, hypoacusia, fatty liver with initial hypertransaminasemia
Previous abortion	No
Previous surgery	Appendectomy
Smoke	No
Gynecological disorders	No
Drugs during pregnancy	Alfa—methyldopa 500 mg 3 times/day, ursodeoxycholic acid 300 mg/day, vitamin D 1,000 UI/day, folic acid 400 mcg/day, acetylsalicylic acid (up to 35 weeks)
Delivery mode	Cesarean section
APGAR 1’	9
APGAR 5’	9
Fetal weight	3,110 g
Neonatal intensive care unit admission	No
Placenta	560 g
Days of maternal hospitalization	6

The patient underwent monthly blood and urinary tests to check hemodynamic, liver, and kidney functions. Monthly maternal echocardiogram always showed normal ejection fraction and no signs of valvulopathy or pulmonary hypertension. During pregnancy, the patient increased by 6 kg in weight; serial blood and urine tests, including full blood count, coagulation profile, LDH, GOT/GPT, γ-GT, alkaline phosphatase, creatinine, haptoglobin, urinalysis, electrolytes, 24 h proteinuria, and urine culture test, were all within the normal range. Screening for fetal chromosomal anomalies showed a minimal risk (<1/100,000 for Down syndrome) and a very low risk (1/1,300) of neural tube defects. The patient was asked to complete a blood pressure diary with daily measurements; her chronic hypertension was successfully controlled with alpha-methyldopa 500 mg 3 times/day. A daily dose of 100 mg acetylsalicylic acid was administered starting from the 10th week of pregnancy in accordance with international guidelines to reduce the risk of preeclampsia (10). In the second trimester, the patient developed gestational diabetes, which was well controlled by a specific diet.

Fetal biometry and umbilical Doppler flowmetry were evaluated monthly. No fetal anomalies were detected at 20 weeks of US screening. Further US scans at 32 and 36 weeks revealed normal fetal growth and normal blood flow in both umbilical arteries.

At 36 weeks, the ratio of soluble fms-like tyrosine kinase 1 (sFlt-1) to placental growth factor (PlGF) (11) showed an increased risk of developing preeclampsia in the following 4 weeks. Due to this and to the initial alteration of some blood pressure values, the patient was hospitalized at 38 weeks to plan labor induction. On admission, a cervical ripening balloon (CRB) was applied for pre-induction, and amniorexis was performed after a few hours. Unfortunately, fetal heartbeat became abnormal due to umbilical cord prolapse, and an urgent cesarean section was needed.

Throughout the pregnancy, the patient's underlying condition remained clinically stable. She continued to experience hypoacusia and reduced vision, which were managed conservatively and did not progress.

A healthy male newborn weighing 3,110 g was born, with APGAR 9/9. The patient and her baby were discharged 4 days after cesarean section, and breastfeeding was carried out. In the first 10 months of life, the baby had normal growth and no signs of Alström disease.

## 3 Discussion

Very little is known about the etiopathogenesis and management of infertility in patients affected by Alström syndrome. The disease is extremely rare (1), with autosomal recessive transmission, and is caused by a series of mutations of the *ALMS1* gene involving different alleles, located on chromosome 2. The protein ALMS is expressed in ciliated cells and contributes to ciliary movement (3). Preliminary studies in the mouse model reported that homozygous mutated ALMS1 females were prone to develop obesity, in turn responsible for a chronic anovulatory state and infertility (12); moreover, the alteration of the *ALMS1* gene was associated with hyperandrogenism and PCOS, with related infertility (13).

In humans, there are very scarce data on the fertility and pregnancy outcome of women affected by AS, likely because of its rarity. Only one pregnancy was previously described, and it was obtained spontaneously (14). Indeed, a careful preconception study aimed at obtaining a well-compensated state of comorbidities is essential for AS patients before planning pregnancy.

We report herein the first live birth obtained after IVF in a woman with AS. The patient we observed had a mild disease form, without relevant heart, kidney, or liver complications, but displayed chronic hypertension and fatty liver disease with increased serum transaminases. The couple was hypofertile due to a relevant oligo-terato-astheno-zoospermia and to the inconsistent presence of ovulation. Moreover, a chronic dysfunction of the tubal ciliated epithelium cannot be ruled out in view of the nature of the syndrome, involving ciliated cells.

ICSI treatment was performed aiming at minimizing all risks, with a mild COS dose, GnRH antagonist protocol with GnRH-agonist trigger, cycle segmentation, and PGT-A. The choice of offering PGT-A despite the young patient's age was discussed with the couple and was accepted by them in view of a likely reduction of the time to live birth and of the risk of miscarriage. Indeed, the patient expressed intense concern about having a miscarriage, and PGT-A is known to significantly reduce the risk of a spontaneous miscarriage (15). During the pretreatment counseling, it was clearly explained to the couple that PGT-A was not useful to avoid AS in their offspring. It was clearly discussed that AS is an autosomal recessive condition, which may be transmitted to the offspring when both parents carry at least one copy of the altered *ALMS1* gene. In our case, the patient was affected by the disease and for sure would have transmitted an altered gene to the fetus, but the husband did not result to carry any of the disease-linked gene variants.

Due to her chronic hypertension, the patient was considered at increased risk for preeclampsia development in pregnancy (16). In the specific case of AS, in addition, there is a dysfunction of ciliated cells: Human chorionic villus mesenchymal stromal cells need a normal function of primary cilia to support placental vascular development (17). Due to limited data, we cannot ascertain whether these patients are indeed at higher risk of hypertensive disorders, but there is biologic plausibility.

The careful pre-conceptional management allowed for the prevention of the development of severe obstetric complications. The pregnancy was strictly followed up by a multidisciplinary team, and the only complication of new onset was gestational diabetes, which was well controlled by a specific diet.

After birth, the newborn was discharged in excellent general conditions, and the tests carried out made it possible to confirm the absence of clinical features potentially attributable to AS.

The successful outcome of this rare case suggests that IVF is feasible in the case of women with AS, provided that they do not have major dysfunction of vital organs and the procedure is carefully planned and performed with all measures to minimize risks. Pregnancy can also be successfully carried out, but a multidisciplinary, continuous follow-up aimed at controlling comorbidity-linked complications is needed.

## Data Availability

The original contributions presented in the study are included in the article/supplementary material, further inquiries can be directed to the corresponding author.
